# Modulation of Burn Hypermetabolism in Preclinical Models

**DOI:** 10.7759/cureus.33518

**Published:** 2023-01-08

**Authors:** Abdullah S Eldaly, Francisco R Avila, Ricardo Torres, Karla Maita, John Garcia, Luiza Serrano, Olivia Ho, Antonio J Forte

**Affiliations:** 1 Plastic Surgery, Mayo Clinic, Jacksonville, USA; 2 Plastic Surgery, Mayo Clinic, Jacksoville, USA

**Keywords:** review, new therapeutics, animal models, hypermetabolism, burn

## Abstract

Severe burns elicit a state of physiological stress and increased metabolism to help the body compensate for the changes associated with the traumatic injury. However, this hypermetabolic state is associated with increased insulin resistance, cardiovascular dysfunction, skeletal muscle catabolism, impaired wound healing, and delayed recovery. Several interventions were attempted to modulate burn hypermetabolism, including nutritional support, early excision and grafting, and growth hormone application. However, burn hypermetabolism still imposes significant morbidity and mortality in burn patients.

Due to the limitations of *in vitro* models, animal models are indispensable in burn research. Animal models provide researchers with invaluable tools to test the safety and efficacy of novel treatments or advance our knowledge of previously utilized agents. Several animal studies evaluated novel therapies to modulate burn hypermetabolism in the last few years, including recombinant human growth hormone, erythropoietin, acipimox, apelin, anti-interleukin-6 monoclonal antibody, and ghrelin therapies. Results from these studies are promising and may be effectively translated into human studies. In addition, other studies revisited drugs previously used in clinical practice, such as insulin and metformin, to further investigate their underlying mechanisms as modulators of burn hypermetabolism.

This review aims to update burn experts with the novel therapies under investigation in burn hypermetabolism with a focus on applicability and translation. Furthermore, we aim to guide researchers in selecting the correct animal model for their experiments by providing a summary of the methodology and the rationale of the latest studies.

## Introduction and background

Severe burns elicit a state of physiological stress and increased metabolism, known as burn-induced hypermetabolism, that may persist for a year after the injury [1]. Initially, the stress response helps the body recover and compensates for the changes associated with the trauma. However, continual hypermetabolism ultimately impairs wound healing, increases infection risk, depletes skeletal muscle mass, and delays rehabilitation [1].

Increased production of inflammatory cytokines and catecholamines mediates burn hypermetabolism [2] and significantly impairs glucose and protein metabolism. Cardiovascular dysfunction, increased lipolysis, insulin resistance/hyperglycemia, skeletal muscle catabolism, and loss of lean body mass (LBM) ensue as a result [3].

The utilization of both non-pharmacological and pharmacological interventions to module burns hypermetabolism had varying levels of success. The current standard of care comprises management through adequate nutritional support. The prescribed diet consists of low-fat, high-carbohydrate enteral feeding alongside energy expenditure through exercise, as overfeeding can accelerate hypermetabolism [4,5]. Failure to intake sufficient calories can also exacerbate hypermetabolism and increase skeletal muscle loss [3]. Early excision and closure of the burn site with skin grafts or other skin substitutes can ameliorate hypermetabolism in severely burned patients [3,6,7]. However, blood loss during the excision of burns of a large surface area remains an obstacle to this practice [8].

Pharmacological interventions include recombinant human growth hormone (rhGH), as it effectively attenuates burn hypermetabolism by increasing insulin-like growth factor-1 (IGF-1) levels [9]. Nevertheless, rhGH use is limited as it increases morbidity and mortality in critically ill patients [10]. Administration of IGF-1, along with its binding protein insulin-like growth factor binding protein-3 (IGFBP-3), reduces muscle catabolism [11]. However, multiple neuropathies associated with IGFBP-3 usage led to discouragement of employment of this combination [3]. In addition, the co-administration of oxandrolone and propranolol diminishes burn hypermetabolism [12]. Finally, metformin application improves glucose levels and muscle protein synthesis in burn patients [13].

Animal models have been essential in burn research for decades. As hypermetabolism still imposes significant morbidity and mortality in burn patients, the need for these models continues to grow. This review aims to provide burn experts and researchers with the latest updates regarding novel burn hypermetabolism modulation therapies with a focus on applicability and translation. Furthermore, we aim to guide researchers in selecting the experimental model most suitable for their hypothesis-driven experiments by providing a brief summary of the latest animal models used in the field.

## Review

Components of burn hypermetabolism

Cardiovascular Dysfunction

Cardiac dysfunction is an expected and well-documented side effect of burn hypermetabolism. Evidence of cardiac dysfunction emerged from animal models and was later supported by human studies.

Adams et al. [14] assessed myocardial contractile changes associated with burn injuries in guinea pigs. Results from their study described, for the first time, contractile dysfunction and impaired diastolic relaxation after a burn. Years later, Maass et al. [15] described the inflammatory cascade that precedes cardiac dysfunction and specified the role played by tumor necrosis factor-alpha (TNF-α) in the inflammatory response by studying a rat model.

In humans, burn injury leads to a rapid drop in heart rate and contractility and a significant decrease in cardiac output (CO). A rebound increase in heart rate and CO follows, and this significantly surpasses the patient's pre-burn baseline resulting in systolic dysfunction [16,17]. Systolic dysfunction increases the number of surgeries, length of stay in the intensive care unit (ICU), and days spent on mechanical ventilation for the patient [18]. Additionally, this cardiovascular response continues beyond recovery of the initial injury with tachycardia and increases resting energy expenditure lasting up to one year after injury [19].

Skeletal Muscle Catabolism

Skeletal muscle catabolism begins immediately after injury and lasts a minimum of nine months in the case of severe burns [20]. Skeletal muscle catabolism is thought to occur due to protein redistribution and the use of amino acids as the energy source [21]. This effect is global and even involves muscles distal to the site of injury. Muscle biopsies from patients with severe burns suggest that satellite cell activation and apoptosis may play a significant role in skeletal muscle wasting [22].

Besides total burn surface area, sepsis, patient weight, and surgical treatment delay increase skeletal muscle catabolism [23]. Furthermore, catabolism and loss of lean body mass (LBM) heighten infection risk, impair wound healing, and delay rehabilitation [23]. In addition, growth restriction in burned pediatric patients may be attributed to this catabolism [3,24].

Insulin Resistance and Hyperglycemia

Insulin resistance (IR) is another metabolic association of severe burns that can persist for up to three years after recovery [16,25,26]. Compensatory hyperinsulinemia develops as an early response to burn-induced hyperglycemia, subsequently followed by lowered insulin levels. IR exists during both the initial hyperinsulinemic and the successive hypoinsulinemic states, which leads to hyperglycemia as a result [27]. IR and hyperglycemia accelerate skeletal muscle catabolism, diminish skin graft survival, and delay wound healing [28,29].

Several factors contribute to IR and hyperglycemia in burn patients. Severe burns induce a rise in glucagon, cortisol, epinephrine, and norepinephrine which leads to lipolysis, proteolysis, and hyperglycemia [27]. The increased level of stress hormones induces inflammatory cytokines and matrix metalloproteinases, causing further IR [30]. Another influential factor is interleukin-1β (IL-1β), distinctively elevated in burn patients, as it is thought to have an apoptotic effect on pancreatic beta cells. This effect inhibits insulin production and leads to hyperglycemia [31]. Likewise, mitochondrial injury in burn patients, particularly in the liver and skeletal muscles, significantly contributes to IR. Mitochondrial injury occurs as a result of increased cytokines and accelerated glycolysis that impair mitochondrial function [32]. The mitochondrial fragments and mitochondrial DNA due to injury lead to a profound inflammatory response and IR [33].

Abnormalities in Lipid Metabolism

The burn-induced rise in catecholamines increases lipolysis leading to a rise in glycerol and free fatty acids (FFA) [34]. Although acute lipolysis is a compensatory response to the injury initially, chronic lipolysis contributes to poorer outcomes in patients [3]. Chronic lipolysis causes a two and a four-fold rise in plasma FFA and glycerol, respectively [35]. Furthermore, adipocytes decrease in size and significantly increase in oxidative capacity, becoming more thermogenic (i.e., producing heat from FFA oxidation) [36,37]. This process is known as the browning of adipose tissue, and it intensifies the expression of uncoupling protein-1 in the adipocyte mitochondria [38]. Browning of white adipose tissue (WAT) plays a role in hypermetabolism and has recently been a focus of burn research.

Animal models in burn hypermetabolism research

Burn research most frequently uses rodents as the preferred animal model. Rats and mice permit a broad range of experimental interventions, are cost-effective, and are easy to house, breed, and handle. Additionally, their formalized pedigree structure and the feasibility of creating various transgenic and knockout strains cause them to be the best fit for many hypothesis-driven experiments [39,40]. However, significant differences between humans and rodents raise questions about their reliability for burn metabolism research.

One fundamental difference is that rodents possess thick fur coat while humans do not. An important explanation of burn hypermetabolism is that the increase in metabolic rate occurs as compensation for the loss of heat through the burned area [41]. This is supported by studies that showed that patients in cooler environments had higher metabolic rates and urinary catecholamine excretion compared to those placed in warmer temperatures [42]. Rodents lose significantly less heat through their skin due to the protection of their fur [43]. This difference in heat loss may compromise the reliability of rodents in burn metabolism research. Furthermore, unlike humans, rodents maintain high levels of activity after the burn and show adaptable metabolic responses and normal eating patterns during the first 24 hours after the injury [40].

Another translational challenge of rodent research is the significant difference in their metabolic profile. These discrepancies affect the ability of rodents to mimic the pathological features of burn hypermetabolism in humans, including insulin resistance, hyperglycemia, and muscle wasting [40]. For example, in contrast to humans, rodents have high levels of high-density lipoprotein (HDL) and low levels of cholesterol and low-density lipoprotein (LDL). In addition, rodents maintain normal serum cholesterol levels even when fed a high-fat diet [44]. These lipid profile disparities are particularly problematic when studying insulin resistance in burn patients [40]. 

Despite the challenges, rodent models play a critical role in our understanding of the effects of catecholamines in burn hypermetabolism [45]. Furthermore, many of the agents currently available for the modulation of burn hypermetabolism were a result of successful translation from rodent models, including recombinant human growth hormone (rhGH) and insulin-like growth factor-1 (IGF-1) [46].

In the following section of this review, we discuss the current therapies for the regulation of burn hypermetabolism in animal models with a focus on applicability and the possibility of translation. Furthermore, we will discuss older therapies being revisited in animal models to better understand their mechanisms and potential. 

Modulation of burn metabolism in animal models

We reviewed the published literature on burn hypermetabolism research in the last 20 years to identify novel innovations in the field. In Table 1, we summarize the most recent animal studies in burn metabolism modulation. For each study, we outline the animal model, the sample size, the described intervention and its rationale, the outcome variables, and the results. In this section of the review, we discuss the most important elements of the studies and elaborate on the results and their relevance to the practice. 

**Table 1:** Summary of burn hypermetabolism modulation in animal models rhHGF - recombinant human hepatocyte growth factor; IL - Interleukin; TNF-α - tumor necrosis factor-alpha; IGF-1 - insulin-like growth factor; mRNA - messenger ribonucleic acid; CRP - C-reactive protein; GHRP - growth hormone releasing peptide; TRH - thyrotropin-releasing hormone; GH - growth hormone; rGH - rabbit growth hormone; GHB - gamma-hydroxybutyrate; DAG - des-acyl-ghrelin; PDC - pyruvate dehydrogenase complex; PDK - pyruvate dehydrogenase kinase; L-NMMA - N(G)-methyl-L-arginine acetate salt; NLRP3 - leucine-rich, repeat-containing family, pyrin-containing 3; WAT - white adipose tissue; LLLT - low-level laser therapy; Pyr-ORS - pyruvate-based oral rehydration salt; WAT - white adipose tissue; PP2A - protein phosphatase 2A; REE - resting energy expenditure; FFA - free fatty acid; EPO - erythropoietin; RBCs - red blood cells

Author and date	Animal model	Number	TBSA (%), burn degree, and type	Intervention	Rationale	Outcome variables	Results	Summary/comments
Jeschke et al. [47] (2000)	Sprague-Dawley rats	56, rhHGF n=28, saline (control) n=28	60%, 3^rd^, scald	rhHGF	HGF stimulates the synthesis of albumin and transferrin and attenuates the synthesis of acute-phase reactants.	Acute phase proteins, cytokines, hepatic gene expression, liver changes, IGF-I concentration were measured one, two, five, and seven days after the burn.	HGF vs. control. Albumin: ↑ (2, 5, 7), transferrin: ↑ (7), α_2_-microglobulin: ↑ (2, 5, 7), IL-6, TNF-α: ↑ (2, 5, 7), liver IGF-I: ↑ (1, 7), serum IGF-I: ↓ (1, 2, 5), hepatic proteins: ↑ (1, 2, 7), liver weight: ↑	HGF appears to modulate the hepatic acute phase response in vivo.
Fang et al. [48] (2002)	Sprague-Dawley rats		30%, 3^rd^, flame	IGF-I	IGF-I is known to reduce burn-induced muscle wasting in rats. The study was conducted to specify the intracellular mechanisms of this effect.	Cathepsin B and L activity, proteasome activity.	Lysosomal-dependent protein breakdown: ↓ by 70%, ubiquitin-dependent protein breakdown: ↓ by 90%, cathepsin B and L activity: ↓, 20S proteasome activity: no effect.	IGF-I down-regulates the activity in the ubiquitin-proteasome pathway by inhibiting steps other than the actual degradation of the substrate in the 20S proteasome.
Banta et al. [49] (2004)	Sprague-Dawley rats	16, rat hindquarters sham n=4, burn n=4, sham+insulin n=4, burn+insulin n=4	20%, 3^rd^, scald	Exogenous insulin	Insulin may have an influence on skeletal muscle proteolysis.	Several metabolic variables, nitrogen balance.	Burn+insulin vs. burn. Nitrogen balance: no change, glucose metabolism: ↑, glutamine, alanine, leucine metabolism: ↓	Although insulin resulted in marked changes in skeletal muscle metabolism, it did not change the nitrogen balance or the rate of muscle proteolysis.
Klein et al. [50] (2004)	Sprague-Dawley rats	64, burn+insulin n=28, burn+NaCl (control) n=28, no intervention (control) n=8	30%, 3^rd^, scald	Exogenous insulin	Insulin has anti-inflammatory effects. The study aimed to evaluate the effect of insulin on liver function, structure, and acute phase response.	Serum glucose, electrolytes, acute phase proteins, hepatic cytokine mRNA, hepatic cytokine protein expression, hepatic morphology, hepatic caspase-3, -9, Bcl-2 expression were measured at days one, two, five, and seven.	Burn+insulin vs. burn. Albumin: ↑, hepatic cytokine mRNA: ↓, TNF-α: ↓, CRP: ↓, Bcl-2:↑, caspase-3,-9: ↓	Insulin attenuates the hepatic inflammatory response, restores hepatic homeostasis, and may improve survival in critically ill subjects.
Weekers et al. [51] (2004)	New Zealand white rabbits	24, saline n=8, GHRP2+TRH n=9, rhGH n=7	15%, 3^rd^	GHRP2, TRH, rhGH	rhGH increased mortality in critically ill patients. The authors hypothesize that this toxicity is explained by the induction of insulin resistance, hyperglycemia, and low thyroid hormone level. Therefore, a combination of GHRP2, TRH, and insulin may result in favorable outcomes.	Serum electrolytes, plasma rGH, plasma IGF-I, plasma TSH, plasma insulin, GH axis, TSH axis, hepatic deiodinase activity.	GHRP2+TRH vs. control. rGH: ↑, TSH: ↑, T_3_:↑, T_4_:↑, type 1 deiodinase: ↑, type 3 deiodinase: ↓	A combination of GHRP2 and TRH reactivated GH and TSH axes and modulated liver deiodinase activity leading to increased T_4_ to T_3_ conversion. Unlike human models, high-dose rhGH therapy was not associated with increased mortality in this rabbit model.
Lang et al. [52] (2007)	Sprague-Dawley rats		40%, 3^rd^, scald	IGF-I	IGF-I increases muscle protein synthesis.	Atrogin-I mRNA, polyUb mRNA, MuRF-I mRNA.	IGF-I therapy. Atrogin-I: ↓ to control levels, PolyUb: ↓ to control levels, MuRF-I: 50% ↓	Burn-induced acute muscle wasting is associated with a glucocorticoid-independent increase in the expression of several Ub E3 ligases which can be downregulated by IGF-I.
Murphy et al. [53] (2007)	Sprague-Dawley rats	60	≥40%, 3^rd^, scald	GHB	GHB increases slow-wave sleep and stimulates endogenous GH secretion.	GH, IGF-I	GHB vs. control. GH: ↑, IGF-I: ↑	GHB increased GH and IGF-I levels which improved wound edge epithelialization and cell layer thickness.
Balasubramaniam et al. [54] (2009)	Sprague-Dawley rats	32, burn+ghrelin n=9, burn+saline n=9, sham+ghrelin n=7, sham+saline n=7	30%, 3^rd^, flame	Ghrelin	Ghrelin synthesis is downregulated after burn injury. The authors hypothesize that ghrelin can attenuate burn-induced skeletal muscle breakdown in rats.	mRNA expression of cytokines, ubiquitin E3 ligases, IGF-I	Ghrelin therapy vs. control. IL-6: ↓, TNF-α: ↓, IGFBP-1: ↓, IGFBP-3: ↓, IGF-I: no effect	Continuous administration of ghrelin significantly inhibited total and myofibrillar protein breakdown in burned rats.
Sheriff et al. [55] (2014)	Sprague-Dawley rats	24, burn n=16, sham n=8	30%, 3^rd^, flame	DAG	Dysregulation of PI3K/Akt signaling plays an important role in skeletal muscle PDC and hyperlactacidemia. The authors hypothesize that DAG can reverse burn-induced skeletal muscle proteolysis through the activation of PI3K/Akt pathway.	mRNA expression of PDC and PDK, skeletal muscle lactate,	DAG ↓ post-burn plasma lactate levels to control levels.	DAG treatment normalized mRnA expression of the PDKs, PDC, and hyperlactacidemia. DAG treatment normalized epinephrine-induced lactate production by isolated skeletal muscles from normal rats.
Chi et al. [56] (2015)	Wistar rats	116	40%, 3^rd^, scald	Apelin, L-NMMA	Previous studies revealed that NLRP3 inflammasome is involved in burn-induced insulin resistance. The authors hypothesize that apelin inhibits NLRP3 and improves insulin resistance in burned rats.	Apelin/APJ mRNA expression in WAT and muscles, plasma apelin level, and activation of the NLRP3 inflammasome in WAT, IL-1 β, IL-6, tumor necrosis factor-α, and monocyte chemoattractant protein-1 levels in plasma, insulin resistance, survival rates, and endothelial nitric oxide synthase phosphorylation in soleus muscles.	Burn+apelin vs. control. Plasma apelin level: ↑, NLRP3 activity: ↓, inflammatory cytokines: ↓, insulin sensitivity: ↑, mortality: ↓	By inhibiting NLRP3 inflammasome, apelin ameliorates insulin resistance and promotes survival after severe burn in rats.
Martins et al. [57] (2015)	Wistar rats	16, burn+ LLLT n=8, burn (control) n=8	45%, 3^rd^, scald	LLLT	LLLT has previously shown positive cellular effect on the wound-healing process.	Tissue morphology, MyoD immunoexpression.	LLLT vs. control. Tissue morphology: more organized, ↑nuclei in fibers, MyoD: ↑	LLLT may modulate burn-induced muscle loss in rats.
Gomez et al. [58] (2018)	Swine	16, limited volume (LV) n=6, modified Brooke (MB) n=6, sham burn n=4	40%, 3^rd^, flame	High vs low volume fluid resuscitation.	The authors hypothesize that fluid volume impacts adrenal response after burn injury.	Urinary cortisol, Gene expression of cleavage enzymes (3β-HSD, CYP17, CYP11, and CYP21), adrenal apoptosis.	MB vs. LV. Urinary Cortisol: ↑, adrenal apoptosis: ↑, adrenal hemorrhage: ↑	High-volume resuscitation was associated with higher cortisol levels and adrenal complications.
Liu et al. [59] (2018)	Sprague-Dawley rats	80, pyr-ORS n=20, sham group n=20, WHO-ORS III n=20, no rehydration n=20	50%, 3^rd^, scald	Pyr-ORS	Pyr-ORS enhances intestinal absorption of water and sodium, improves visceral blood flow, and maintains organ function in animal models.	Mortality, systemic hemodynamics, lactic acidosis.	Pyr-ORS vs. WHO-ORSIII. Mortality:↓, lactic acidosis:↓, systemic hemodynamics: ↑	Pyruvate corrected hypoxic lactic acidosis and improved survival in rats subjected to lethal burn shock.
Auger et al. [60] (2019)	C57BL/6 mice		30%, 3^rd^, scald	Metformin	Preservation of WAT improves outcomes in hypermetabolic states. The authors hypothesize that metformin may prevent the browning of subcutaneous WAT.	Blood glucose, plasma cortisol, plasma adrenaline, PP2A.	Metformin vs. control. Lipolysis: ↓, PP2A: ↑	Metformin reduces lipotoxicity associated with burn in rats by inhibiting pathological browning of WAT.
Yang et al. [61] (2019)	Sprague-Dawley rats	30, burn n=10, burn+glutamine n=10, control n=10	30%, 3^rd^, scald	Glutamine	Glutamine is an important component of the nutritional support of burn patients. The authors used a rat model to study the underlying mechanisms.	Alanine, histidine, creatinine, REE, lactic acid.	Burn+glutamine vs. burn. Alanine: ↓, leucine: ↓, lactic acid: ↓, NADPH: ↑, reduced glutathione: ↑	Glutamine reduces oxidative stress, ↑ATP synthesis, ↓ hypermetabolism after burn injury possibly through increasing reductive compounds such as NADPH and reduced glutathione.
Barayan et al. [62] (2020)	C57BL/6J mice		30%, 3^rd^, scald	Acipimox	Acipimox inhibits lipolysis by suppressing hormone-sensitive lipase. The authors hypothesize that inhibiting burn-induced lipolysis can prevent organ damage and improve survival.	Adipose tissue histology, plasma FFA, plasma glycerol, liver triglycerides.	Acipimox vs. control. Lipolysis: ↓, FFA: ↓, liver weight: ↓, hepatic intracellular fat: ↓	Acipimox suppresses burn-induced lipolysis and may improve burn function after the burn.
Wu et al. [63] (2020)	Sprague-Dawley rats	24, sham-control n=6, burn n=6, burn+weekly EPO n=6, burn+daily EPO n=6	Hind-limb, 3^rd^, flame	EPO	The authors hypothesize that EPO may reduce postburn muscle atrophy.	Histological analysis, RBC mass, ubiquitin ligase, atrogin-1, caspase 3.	EPO vs. burn only. Ubiquitin ligase: ↓, atrogin-1: ↓, caspase 3: ↓, myofiber cross section area: ↑	EPO attenuated burn-induced skeletal muscle loss. No significant difference between daily and weekly EPO. However, daily EPO resulted in marked erythrocytosis. Therefore, weekly EPO might be a safer option in burn patients.
Barayan et al. [64] (2021)	C57BL/6J mice		30%, 3^rd^, scald	Anti-IL-6 monoclonal antibody	IL-6 is a major contributor to burn hypermetabolism. Pharmacological blockade of IL-6 may ameliorate burn-induced hypermetabolism.	Histological analysis, Inflammation, and organ damage biomarkers.	Anti-IL-6 vs. control. Lipolysis: ↓, hepatic lipotoxicity: ↓, browning-induced lipolysis: ↓, inflammation biomarkers: ↓	Daily anti-IL-6 therapy protects against burn-induced weight loss without adverse effects on mortality.

Recombinant Human Hepatocyte Growth Factor (rhHGF)

The liver plays a vital role in the burn-induced systemic inflammatory response by producing acute-phase proteins [47]. In patients with severe burns, up-regulation of acute-phase proteins in the liver occurs, while the production of constitutive proteins, such as albumin and transferrin, is down-regulated [65,66]. This shift in protein presentation stimulates immune function, coagulation, and wound healing [66,67]. Despite the imperative role of acute-phase proteins in the initial post-burn phase, a prolonged rise of these protein levels is associated with increased mortality [68]. Moreover, this stress response ultimately heightens the metabolic rate [20].

HGF improves hepatic function, stimulates constitutive hepatic protein synthesis, and reduces the synthesis of acute-phase proteins in animal models and humans [69-71]. In human hepatocytes, HGF stimulates the synthesis of albumin, fibronectin, and transferrin while decreasing the synthesis of haptoglobin and alpha-1 antichymotrypsin [70]. Therefore, patients with severe burns may benefit from HGF therapy that lessens the burn-induced inflammatory and metabolic response.

Jeschke et al. [47] investigated the effect of rhHGF on acute-phase proteins, constitutive hepatic proteins, IGF-1, and inflammatory cytokines in thermally injured rats. rhHGF successfully stimulated constitutive protein production as it normalized serum transferrin levels seven days after injury and attenuated the decrease in serum albumin. In contrast to data from in vitro studies, rhHGF increased acute-phase proteins, including α2-macroglobulin and TNF-α. The stimulatory effect of rhHGF on interleukin 6 (IL-6) explains the event as induction of IL-6 results in escalated levels of its dependent proteins, α2-macroglobulin and TNF-α. Surprisingly, the increase in α2-macroglobulin and TNF-α, known for their catabolic effects, was not related to weight loss in the treated rats. Finally, rhHGF lowered serum IGF-1 and expanded hepatic IGF-1 levels, suggesting a possible role of IGF-1 in activating transcription factors during the acute-phase response.

Despite the promising results reported by Jeschke et al. [47], conventional methods pose difficulty in achieving the required serum concentrations. Effective HGF delivery to the target organs remains an obstacle for successful translation due to the rapid elimination of HGF from the body after intravenous (IV) injection [72]. Several strategies were developed for adequate HGF delivery with variable success. Ozeki et al. [73] proposed a controlled HGF release by using biodegradable gelatin hydrogel. This method achieved sustained HGF levels and was successfully utilized in animal models of liver cirrhosis and arthritis [74,75]. A major limitation is purifying HGF in its heterodimeric biologically active form [76]. Other approaches for delivery include IV delivery of the HGF gene using a hydrodynamics-based transfection system [77] and genetically engineered mesenchymal stem cells that can express HGF [78]. KP-100 (rhHGF, Kringle Pharma, Inc.) is in multiple clinical trials that evaluate HGF efficacy in amyotrophic lateral sclerosis, spinal cord injuries, and vocal fold scars [79-81]. However, this has not yet been investigated in burn patients.

Recombinant Human Growth Hormone (rhGH)

In rat models, rhGH decreased both IL-1 expression and type-1 acute phase protein levels and regulated the hepatic acute phase response to burn. On the other hand, rhGH increased hepatic mitosis and total hepatic protein concentrations [46]. This effect was explained by IGF-1 stimulation in the hepatic parenchyma [9]. In human studies, severe burns demonstrated a decrease in IGF-1 levels, thought to play a role in burn catabolism, and was considered a target of hypermetabolism modulation therapies [82]. 

In a randomized clinical trial, an intramuscular injection of 0.2 mg/kg/day for one year noticeably improved the outcomes of severely burned pediatric patients by preserving lean body mass and attenuating burn hypermetabolism. In those patients, rhGH therapy significantly increased serum growth hormone, IGF-1, and IGFBP-3 [83]. In the same patient population, rhGH markedly reduced serum levels of TNF-α, IL-1, and C-reactive protein (CRP) [84].

Although rhGH improved outcomes without increasing mortality in pediatric patients, it was associated with increased hyperglycemic episodes, insulin resistance, morbidity, and mortality in non-burned critically ill adult patients. Moreover, rhGH prolonged the length of ICU stay and days on mechanical ventilation [10]. Although the study was done on patients without a burn injury, the findings can limit the application of rhGH therapy in burned adult patients.

Weeker et al. [51] hypothesized that the increased mortality from rhGH is a direct result of insulin resistance, hyperglycemia, and low thyroid hormone levels in critically ill patients. Therefore, a combination of GHRP and TRH was tested in a rabbit model to mitigate the adverse effects of rhGH. The combination reactivated GH and TSH axes leading to increased T4 to T3 conversion. However, the rhGH therapy alone did not increase mortality in rabbit models, unlike it did in humans, which renders the translational ability of the model questionable.

Insulin-Like Growth Factor-1 (IGF-1)

Muscle wasting after a burn injury ensues due to increased protein breakdown and decreased muscle protein synthesis. IGF regulates many anabolic and catabolic pathways [85]. Evidence from human studies suggests a beneficial effect of IGF-1 combined with IGFBP-3 in lowering muscle catabolism and improving immune function in burn patients [11,86]. However, the development of neuropathies associated with IGFBP-3 therapy limited its clinical use [3].

Recent animal studies evaluated pathways influenced by IGF-1. IGF-1 was found to stimulate protein synthesis and attenuate skeletal muscle catabolism in rats [87,88]. Fang et al. [48] investigated the mechanisms by which IGF-1 inhibits muscle catabolism in burned rats. They showed that IGF-1 reduces muscle wasting by restricting lysosomal and ubiquitin-proteasome-dependent protein degradation. Moreover, their results demonstrated that IGF-1 does not affect the calcium-calpain-dependent pathway.

Lang et al. [52] studied skeletal muscle catabolism pathways in rats and the influence of IGF-1. The data from their experiment suggest that glucocorticoid-independent increased expression of Ub E3 ligases prompts burn-induced muscle catabolism. Additionally, IGF-1 effectively downregulated Ub E3 ligases and, thus, attenuated muscle wasting.

Insulin

**Figure 1:**
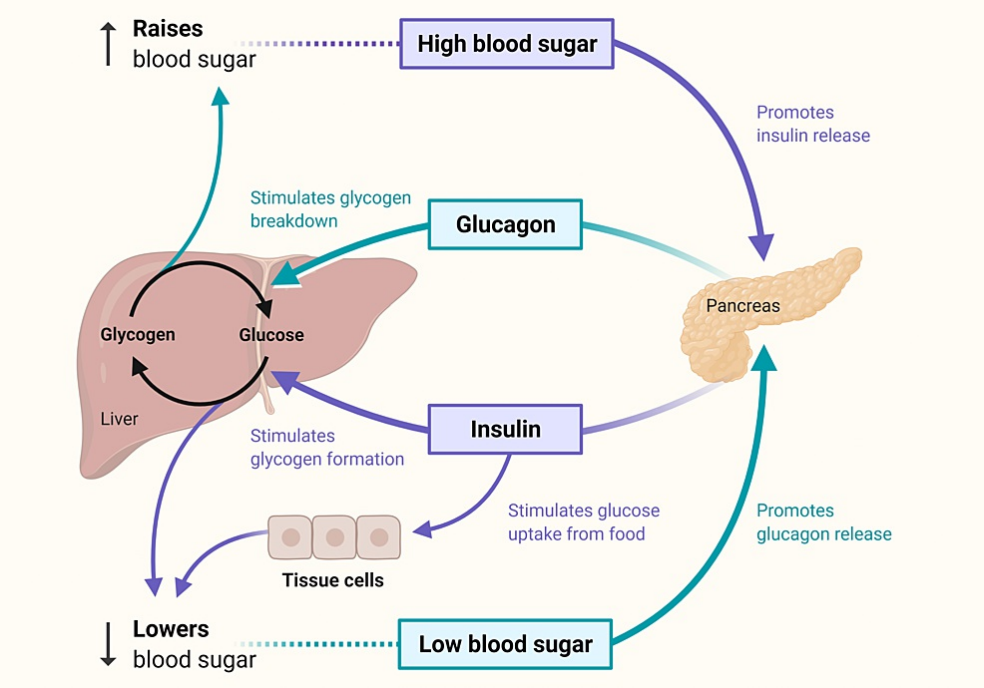
Glucose control under normal conditions

Under normal conditions, glucose levels are tightly controlled by the complementary actions of glucagon and insulin (see Figure 1) [89]. Insulin resistance and the resulting hyperglycemia are the hallmarks of burn hypermetabolism and are associated with muscle breakdown and impaired wound healing [26,28]. The burn-induced rise in catecholamines inhibits insulin release and glucose uptake [90,91]. Furthermore, increased cortisol and catecholamine levels further hinder the anabolic effect of insulin.

The prevailing treatment of burn-induced hyperglycemia is insulin therapy, and it demonstrated favorable outcomes as it reduces muscle wasting and improves wound healing [92,93]. Unfortunately, maintaining this hyperinsulinemic-euglycemic state is delicate, as it was associated with a significant increase of severe hypoglycemic episodes [94]. Therefore, close monitoring of burn patients on insulin therapy is recommended to avoid adverse events.

Although insulin is a well-established treatment, it is frequently revisited in animal studies to expand the knowledge of the underlying mechanisms and investigate further potential applications. In a study done by Banta et al. [49], they used a perfused rat hindquarter preparation to study the effect of insulin on skeletal muscle metabolism in burned rats. Significant metabolic changes in the muscles followed exogenous insulin application. First, glucose metabolism markedly increased in the muscle. Then, increased flux through the pentose phosphate pathway occurred. Finally, the metabolism of glutamine, alanine, and leucine decreased. Surprisingly, the nitrogen balance and the rate of protein proteolysis in the muscle remained unchanged.

Klein et al. [50] evaluated the anti-inflammatory response of insulin in burned rats. Exogenous insulin reduced serum concentration of TNF-α, C-reactive protein (CRP), and hepatocyte cytokine mRNA transcription. The findings from this study suggest a robust anti-inflammatory role of insulin in burned rats that can diminish morbidity and mortality.

Ghrelin

Ghrelin is a gastrointestinal peptide connected to various biological processes [95], including energy and glucose homeostasis, enhancement of growth hormone (GH) release, and modulation of the inflammatory and metabolic pathways [96]. Its physiological role establishes it as a potential therapeutic agent for burn hypermetabolism.

A benefit of ghrelin is that it enhances GH and IGF-1 release, improving skeletal muscle catabolism [11,86,97]. Additionally, ghrelin stimulates lipid storage in adipocytes via a SIRT1/p53/AMPK pathway, which may positively affect burn hypermetabolism by inhibiting the browning of WAT [98]. Moreover, ghrelin shows anti-inflammatory functions through the induction of IL-10 expression and reduction of IL-1β, IL-6, and TNF-α [99].

Serum ghrelin levels drop initially after a burn injury before rising again two to three weeks later [100]. In a rat model [54], continuous ghrelin administration attenuated burn-induced skeletal muscle breakdown through multiple mechanisms. In one process, ghrelin impaired the inhibition of mRNA expression of IGFBP-I and IGFBP-III. In another, ghrelin inhibited increased mRNA expression of binding proteins in the skeletal muscle. Additionally, ghrelin significantly reduced TNF-α and IL-6 mRNA expression in skeletal muscles. Finally, ghrelin lessened the development of plasma glucocorticoids that arise after a burn. In other animal models, ghrelin therapy improved burn-induced impaired gastrointestinal motility and wound healing [95,101,102].

Anamorelin, an oral mimetic of ghrelin, was tested in patients with cancer-related cachexia [103]. In those patients, anamorelin significantly increased GH, IGF-1, and IGFBP-3. These results indicate that ghrelin may be a promising therapy in burn hypermetabolism. However, hyperglycemia can be a potential side effect of ghrelin use which will then require close monitoring of blood glucose levels and adjustment of the insulin therapy accordingly. 

Metformin

Initially introduced as an oral hypoglycemic agent for type II diabetes mellitus, metformin is a biguanide drug that stimulates peripheral glucose uptake and inhibits hepatic glucose production. Over the years, the expansion of metformin use occurred to include it in the treatment of multiple cancers, obesity, liver diseases, cardiovascular diseases, and renal diseases [104]. In addition, metformin effectively weakened the inflammatory response and reduced insulin resistance in burn patients [105]. However, there was a lack of understanding of numerous metformin pathways, and thus, close investigation of these mechanisms through animal models transpired.

**Figure 2:**
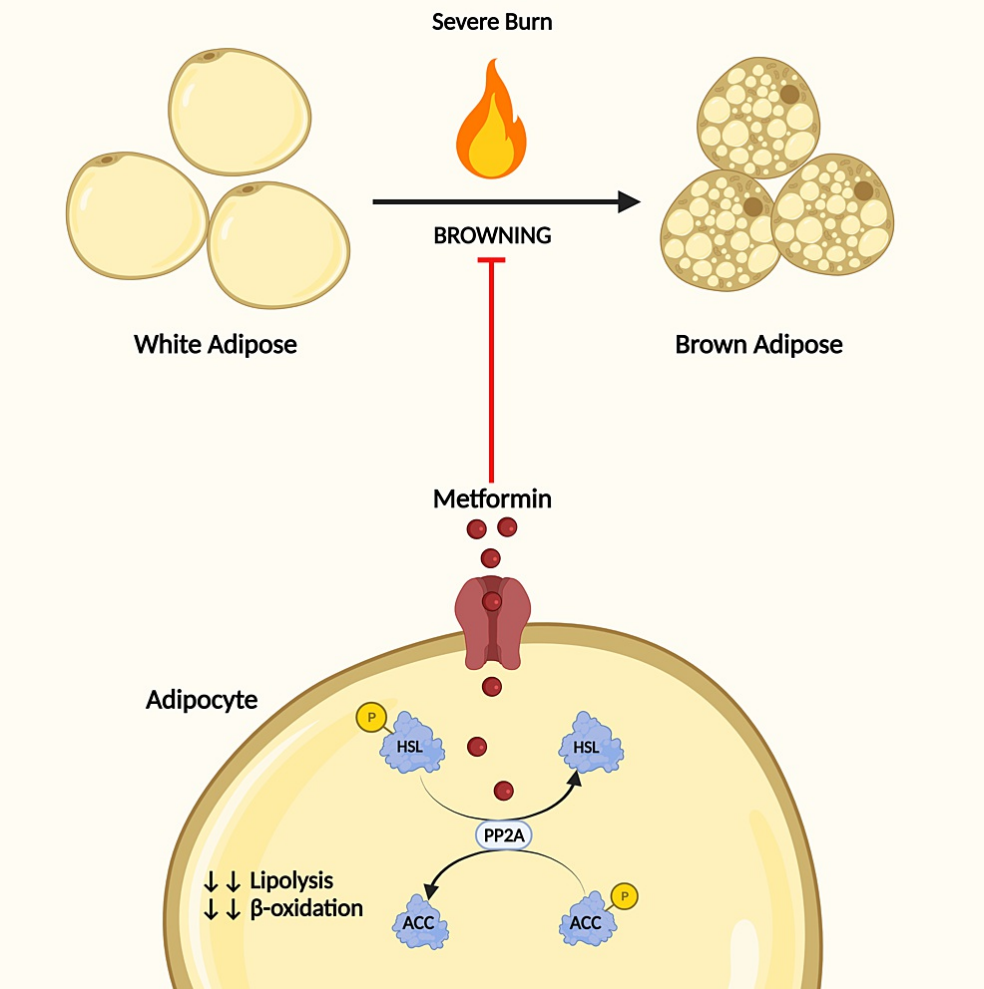
Metformin effect on burn-induced browning of adipose tissue Severe burn leads to the browning of white adipose tissue. Metformin promotes the whitening of adipose tissue by inducing protein phosphatase 2A (PP2A). PP2A then catalyzes acetyl-CoA carboxylase (ACC) and hormone-sensitive lipase (HSL), enhancing fat storage and inhibiting lipolysis.

Browning of WAT occurs in critically ill patients, such as severely burned patients. Auger et al. [60] explored the effect of metformin on WAT and its underlying mechanism in a burned mouse model and human ex vivo adipose tissue. Metformin promoted the whitening of adipose tissue by inducing protein phosphatase 2A (PP2A). PP2A then catalyzed acetyl-CoA carboxylase and hormone-sensitive lipase, enhancing fat storage and inhibiting lipolysis (see Figure 2). Furthermore, metformin appears to improve survival in the treated animals, as the treated cohort showed 100% survival compared to 80% survival in the untreated animals. Finally, metformin significantly attenuated the weight loss in the treated versus untreated animals.

In a randomized clinical trial (RCT), Jeschke et al. [105] proved that metformin was as effective as insulin in controlling burn-induced hyperglycemia without causing hypoglycemia. Furthermore, metformin improved insulin resistance and reduced endogenous insulin production. Thus, this indicates a superior safety profile than insulin for burn patients.

Apelin

**Figure 3:**
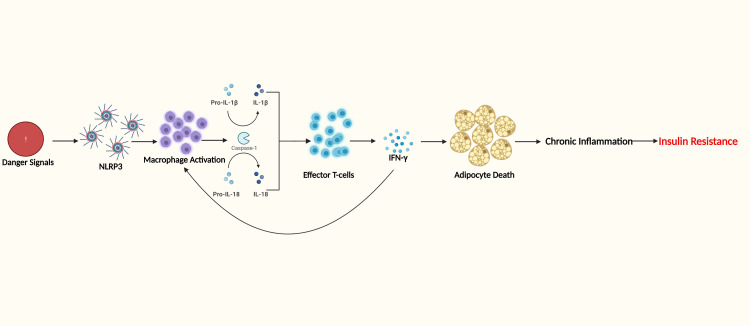
NLRP3 inflammasome role in burn hypermetabolism Danger signals released by adipocyte death include hypoxia, necrosis, and reactive oxygen species. These signals are sensed by NLRP3 inflammasome that activates a pro-inflammatory sequence of events. This sequence leads to chronic inflammation and insulin resistance. NLRP3 - nucleotide-binding domain and the leucine-rich, repeat-containing family, pyrin-containing 3; IL - interleukin; IFN-Ɣ - interferon-Ɣ

Growing evidence from the literature suggests a strong link between inflammation and increased insulin resistance, obesity, and type II diabetes mellitus [106]. Recent studies show the role of IL-Iβ and IL-18 in developing insulin resistance [107,108]. A multi-protein complex, termed the inflammasome, activates IL-Iβ [108]. The nucleotide-binding domain and the leucine-rich, repeat-containing family, pyrin-containing 3 (NLRP3) inflammasome, mediate obesity-induced inflammation and insulin resistance (see Figure 3) [108]. Moreover, NLRP3 inflammasome was activated in the WAT of burn patients, indicating a possible function in conciliating burn-induced insulin resistance [109].

Apelin is a peptide hormone that serves as the endogenous ligand for the G-protein coupled receptor APJ and plays variable anti-inflammatory and cell regulatory roles [110,111]. A significant decrease in skeletal muscle's apelin/APJ expression was noticed in animal models with severe insulin resistance [112]. Although apelin increases insulin resistance and appears to inhibit pancreatic insulin secretion [113], insulin sensitivity in animal models improved following apelin therapy [114]. Assuming that apelin exerts different regulatory roles according to insulin resistance levels can explain this contradiction. Because apelin appears to correct insulin sensitivity in conditions of severe insulin insensitivity, it may assist in the attenuation of burn-induced insulin resistance.

Chi et al. [56] investigated apelin's effects on insulin resistance in a rat model. Their results showed that apelin/APJ expression markedly decreased in the skeletal muscles after a burn injury. On the other hand, apelin/APJ expression in the WAT and plasma apelin levels remained unchanged after injury. Apelin treatment failed to change apelin/APJ expression levels in both skeletal muscles and WAT. However, apelin therapy successfully prevented NLRP3 inflammasome activation in the WAT. Moreover, apelin weakened the burn-induced elevation of the inflammatory cytokines IL-Iβ and IL-6. Finally, the therapy adequately helped treat burn-induced insulin resistance in the animals. These findings suggest that apelin can be an effective agent in combating insulin resistance in burn patients.

Although apelin is being heavily investigated for its potential therapeutic effects in heart failure and pulmonary hypertension, no clinical trials have taken place. One crucial challenge to translation is the lack of a potent and selective drug that can efficiently target the apelin/APJ signaling pathway in humans [115]. 

Erythropoietin (EPO)

Besides its primary function as a potent stimulant of erythropoiesis, EPO has significant anti-inflammatory and anti-apoptotic functions [116]. Evidence from animal studies imply that EPO can reduce apoptosis and inhibit inflammatory cytokines production in sepsis [117] and ischemic/reperfusion injuries [118]. In humans, EPO elevates the expression of myogenic differentiation factor in satellite cells leading to a significant improvement in muscle regeneration of healthy young men [119]. These findings indicate a possible role of EPO in modulating burn metabolic response, particularly in skeletal muscles.

**Figure 4:**
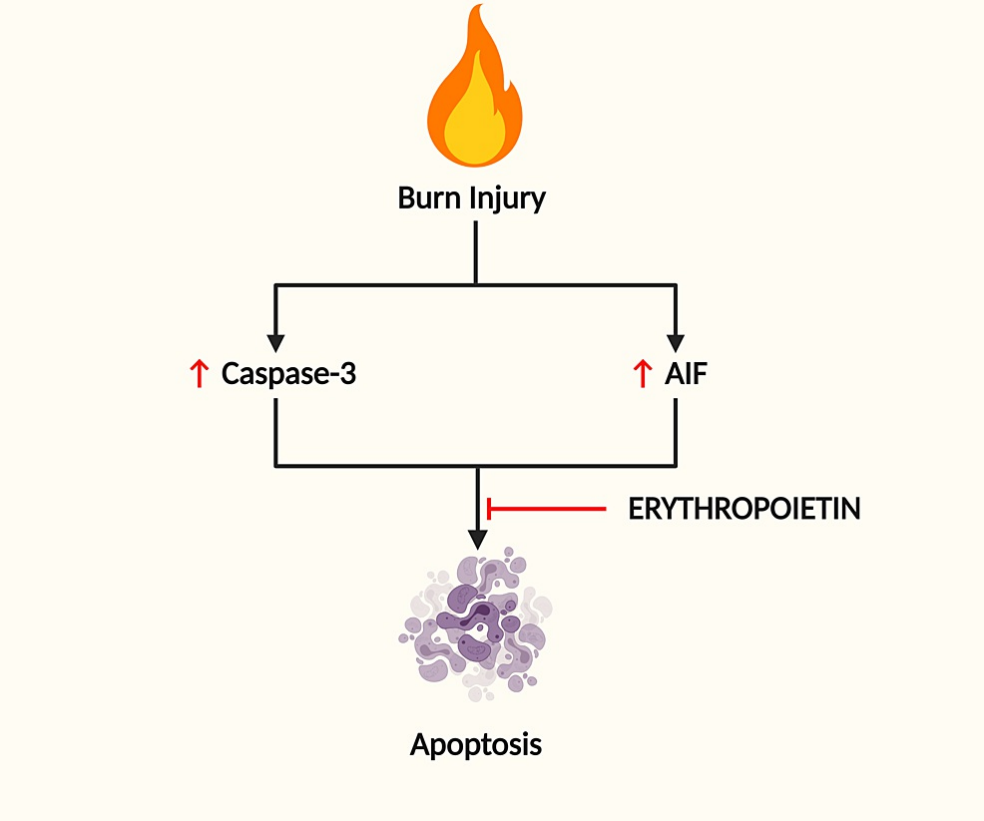
Erythropoietin inhibits burn-induced apoptosis Erythropoietin (EPO) decreased the expression of cleaved caspase-3 in the first mechanism. In the second, EPO attenuated the formulation of apoptosis-inducing factor (AIF) in skeletal muscles.

Wu et al. [63] assessed the effects of EPO on caspase-dependent and caspase-independent apoptosis in the skeletal muscles of a rat model. The results demonstrated that EPO prevented apoptosis in skeletal muscles by two mechanisms. EPO decreased the expression of cleaved caspase-3 in the first mechanism. In the second, EPO attenuated the formulation of apoptosis-inducing factor (AIF) in skeletal muscles (see Figure 4). Additionally, EPO lessened muscle fibrosis by impeding both tissue-growth facto β (TGF-β) and connective tissue growth factor (CTGF), which are believed to be involved in burn-induced muscle fibrosis.

Li et al. [120] showed that EPO could improve functional recovery in critically ill trauma patients. Another study done by Günter et al. [121] revealed the regenerative effects of EPO in burn patients and its good safety profile. However, EPO's possible role in burn hypermetabolism is yet to be investigated in humans.

Anti-IL-6 Monoclonal Antibody

IL-6 is a pleiotropic cytokine that regulates immune and metabolic functions in both normal and stress conditions [64]. IL-6 levels are notably heightened after a burn injury, and the magnitude of elevation correlates to the severity of the injury, organ dysfunction, and poorer outcomes [122,123]. Data from animal studies suggest that IL-6 plays a role in the browning of the WAT, a major adverse event of burn hypermetabolism [124]. WAT browning caused hepatic steatosis and dysfunction and was associated with increased morbidity and mortality in burned mice [62]. Moreover, WAT browning was linked to persistent hypermetabolism and poor long-term outcomes [124]. Therefore, blocking the IL-6 pathway can decrease WAT browning and prevent its unfavorable consequences.

Barayan et al. [64] examined the effects of anti-IL-6 monoclonal antibody (mAb) therapy on post-burn metabolism in a mouse model. The findings from the study imply that daily anti-IL 6 mAb can modulate several post-burn metabolic changes. Anti-IL 6 mAb lowered burn-induced lipolysis and WAT browning. Moreover, anti-IL 6 mAb inhibited the development of post-burn hepatic steatosis. Finally, blocking IL-6 signaling had anti-fibrotic effects on the skin.

Tocilizumab, a humanized anti-IL-6 receptor antibody, is used as an effective treatment for autoimmune conditions such as rheumatoid arthritis, giant cell arteritis, and juvenile idiopathic arthritis [125]. Tocilizumab has not been previously investigated in burn patients. However, one of the limitations of tocilizumab use in burn patients may be the increased risk of infections, particularly in the skin and the respiratory tract [126].

Acipimox

Acipimox is a nicotinic acid analog and an approved anti-lipolytic drug that exerts its action by inhibiting hormone-sensitive lipase (HSL) [62,127]. Acipimox may play a role in balancing the burn metabolic response, particularly WAT browning, due to its effectiveness in suppressing lipolysis.

Barayan et al. [62] considered the effect of acipimox therapy on WAT browning in a mouse model, and the findings illustrate that it ameliorates burn hypermetabolism via several pathways. In the treated animals, acipimox prevented burn-induced weight loss and suppressed WAT browning. Furthermore, by inhibiting lipolysis, acipimox reduced plasma-free fatty acids. Finally, acipimox hindered hepatic fat infiltration.

In patients with bulimia nervosa, acipimox showed metabolic effects that can be applicable to burn patients. In a randomized clinical trial, Smitka et al. [128] investigated the effect of acipimox combined with short-term physical exercise on the metabolism of bulimic and healthy-weight women. In both groups, a combination of acipimox therapy and a short-term physical exercise program successfully induced GH levels and reduced peripheral lipolysis. However, the effect on peripheral lipolysis was more profound in bulimic patients. In another randomized crossover study [129], acipimox improved insulin resistance and increased glucagon-like peptide-1 (GLP-1) levels in hypopituitary patients. Furthermore, acipimox inhibited lipolysis by activating a PUMA-G receptor-dependent pathway [129]. To date, acipimox, as a modulator of burn hypermetabolism, has not been studied in humans. However, data from clinical trials on acipimox use in other conditions establish the feasibility for future clinical trials in burn patients to explore possible benefits.

Glutamine

**Figure 5:**
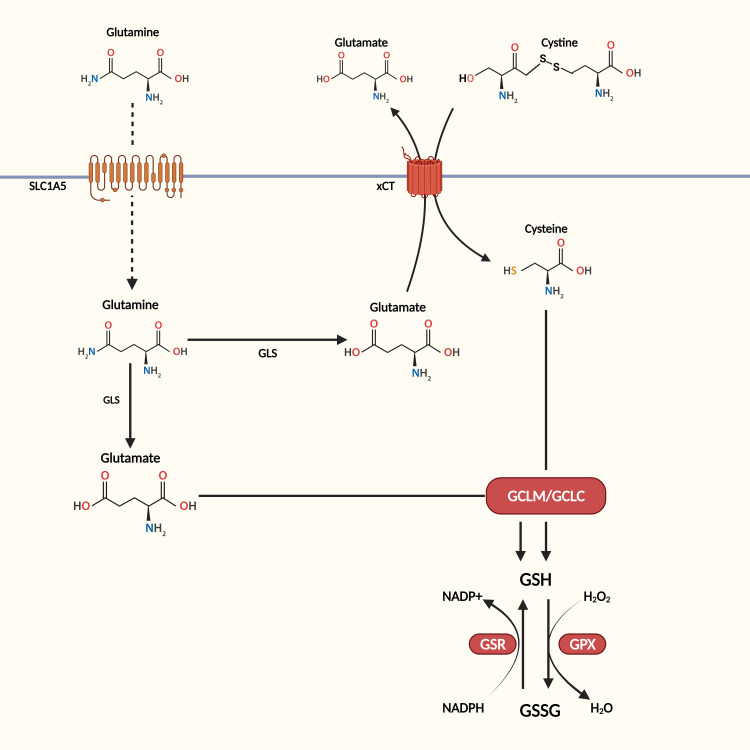
The role of glutamine in regulating oxidative stress GSH - reduced glutathione; GCLM - glutamate-cysteine ligase modifier subunit; GCLC - glutamate-cysteine ligase catalytic subunit; GPX - glutathione peroxidase; GSR - glutathione reductase; GSSG - oxidized glutathione

Glutamine is the most abundant amino acid in the human body and performs multiple functions in the state of health and disease [130]. Glutamine converts into various amino acids and plays a vital role in the tricarboxylic acid cycle [131]. Lymphocyte proliferation, macrophage functions, and neutrophil bacterial killing require glutamine [130]. In addition, glutamine regulates oxidative stress as it contributes to glutathione production (see Figure 5). Glutamine becomes an essential amino acid during hypercatabolic states, and its supplementation may prevent gut barrier failure [132]. Although glutamine therapy in burn patients significantly ameliorated proteolysis and regulated burn metabolism [133], the underlying mechanisms of these effects have not been explored.

Yang et al. [61] investigated the effects and the underlying mechanisms of glutamine therapy in a rat model. Glutamine therapy significantly ameliorated burn-induced weight loss and reduced resting energy expenditure. Furthermore, glutamine therapy reversed the burn-induced decline in urea, glutamine, glutathione, and α keto-glutarate levels. Contrarily, glutamine lowered the levels of creatinine, lactic acid, succinic acid, and histidine. Additionally, glutamine therapy was associated with enhanced bile acid metabolism, leading to improved liver functions and better absorption of nutrients. Finally, increased synthesis of reductive substance due to glutamine employment alleviated post-burn oxidative stress, recovered mitochondrial function, and promoted anabolism. The authors proposed nine pathways where glutamine exerts its effect in the burned rats, which include alanine synthesis, leucine synthesis, isoleucine synthesis, Krebs cycle, and glutathione metabolism.

## Conclusions

Animal models are the foundation of burn research. In this review, we explored the most recent advances in burn metabolism research. Several studies evaluated novel therapies, including rhHGF, EPO, acipimox, apelin, anti-IL-6 mAb, and ghrelin. Other studies revisited drugs previously used in clinical practice, such as insulin and metformin, to deepen the understanding of underlying mechanisms. Ultimately, we believe that the growing evidence from animal studies urges further clinical research to establish practical guidelines to mitigate burn hypermetabolism. 
